# Reliability and application of the new morphological classification system for chronic symptomatic osteoporotic thoracolumbar fracture

**DOI:** 10.1186/s13018-020-01882-5

**Published:** 2020-08-24

**Authors:** Ding-Jun Hao, Jun-Song Yang, Yuan Tuo, Chao-Yuan Ge, Bao-Rong He, Tuan-Jiang Liu, Da-Geng Huang, Shuai-jun Jia, Peng Liu, Jia-Nan Zhang, Jin-Peng Du

**Affiliations:** Department of Spine Surgery, Honghui Hospital, Xi’an Jiaotong University, No 555, Friendship Rd, District Beilin, Xi’an, 710054 China

**Keywords:** Osteoporotic, Thoracolumbar, Fractures, Spine, Morphology, Classification, Reliability, Kümmell’s disease

## Abstract

**Background:**

We propose a new classification system for chronic symptomatic osteoporotic thoracolumbar fracture (CSOTF) based on fracture morphology. Research on CSOTF has increased in recent years; however, the lack of a standard classification system has resulted in inconvenient communication, research, and treatment. Previous CSOTF classification studies exhibit different symptoms, with none being widely accepted.

**Methods:**

Imaging data of 368 patients with CSOTF treated at our hospital from January 2010 to June 2017 were systematically analyzed to develop a classification system. Imaging examinations included dynamic radiography, computed tomography scans, and magnetic resonance imaging. Ten investigators methodically studied the classification system grading in 40 cases on two occasions, examined 1 month apart. Kappa coefficients (*κ*) were calculated to determine intraobserver and interobserver reliability. Based on the radiographic characteristics, the patients were divided into 5 types, and different treatments were suggested for each type. Clinical outcome evaluation included using the visual analog score (VAS), the Oswestry disability index (ODI), and the American Spinal Injury Association (ASIA) impairment scale.

**Results:**

The new classification system for CSOTF was divided into types I–V according to whether the CSOTF exhibited dynamic instability, spinal stenosis or kyphosis deformity. Intra- and interobserver reliability were excellent for all types (*κ* = 0.83 and 0.85, respectively). The VAS score and ODI of each type were significantly improved at the final follow-up compared with those before surgery. In all patients with neurological impairment, the ASIA grading after surgery was significantly improved compared with that before surgery (*P* < 0.001).

**Conclusions:**

The new classification system for CSOTF demonstrated excellent reliability in this initial assessment. The treatment algorithm based on the classification can result in satisfactory improvement of clinical efficacy for the patients of CSOFT.

## Background

As early as 1891, Kümmell et al. noticed that some middle-aged and elderly patients would gradually develop back pain and progressive kyphosis after minor trauma followed by an asymptomatic period lasting weeks to months. Due to limited availability of radiological imaging technology, Kümmell’s was limited to symptom observation alone. However, the original intention of his report on this phenomenon was to distinguish it from Pott’s kyphosis in spinal tuberculosis [1]. As radiological examination technology became more available, and subsequent scholars confirmed Kümmell’s disease to be old osteoporotic thoracolumbar fractures (OTF). In some patients, ischemic osteonecrosis or nonunion of the vertebral body will occur, which manifested together with the intravertebral cleft (IVC) or *intravertebral vacuum phenomenon* [2–7]. Thus, the definition of this type of OTF is widely divergent. In honor of Kümmell’s contributions, early scholars used the term “Kümmell’s Disease”, followed by a series of descriptive factors reflecting various pathological changes of OTF, such as vertebral ischemic osteonecrosis or vertebral nonunion [1, 5, 6]. These names address the symptoms or pathological changes of patients with OTF, but they cannot fully summarize the imaging characteristics. Due to the distinct differences in fracture morphology and patient symptoms, the surgical algorithm remains controversial [8–12]. Thus, we proposed a new concept of classifying chronic symptomatic osteoporotic thoracolumbar fractures (CSOTF). Based on the imaging characteristics of 368 CSOTF patients, we designed a novel classification system that divides these patients into five types. We evaluated the reliability of this classification system and subsequently proposed a treatment algorithm.

## Methods

The Ethics Committee of Honghui Hospital approved this study. We retrospectively collected 6758 cases of OTF in our hospital from January 2010 to August 2017. The following diagnostic criteria for CSOTF were used: (1) supine position computed tomography (CT) scan or magnetic resonance imaging (MRI) exhibited signs of an IVC, (2) bone mineral density was less than or equal to − 2.5 standard deviations (SD), and (3) exclusion of vertebral collapse caused by tumor or infection. A total of 483 (7.1%) patients were diagnosed with CSOTF after screening. We excluded cases involving multiple CSOTF or with incomplete imaging data to ensure the accuracy of the classification system. The imaging data of 368 patients of were ultimately used to develop the new classification system.

### Radiological measurement

**Fig. 1:**
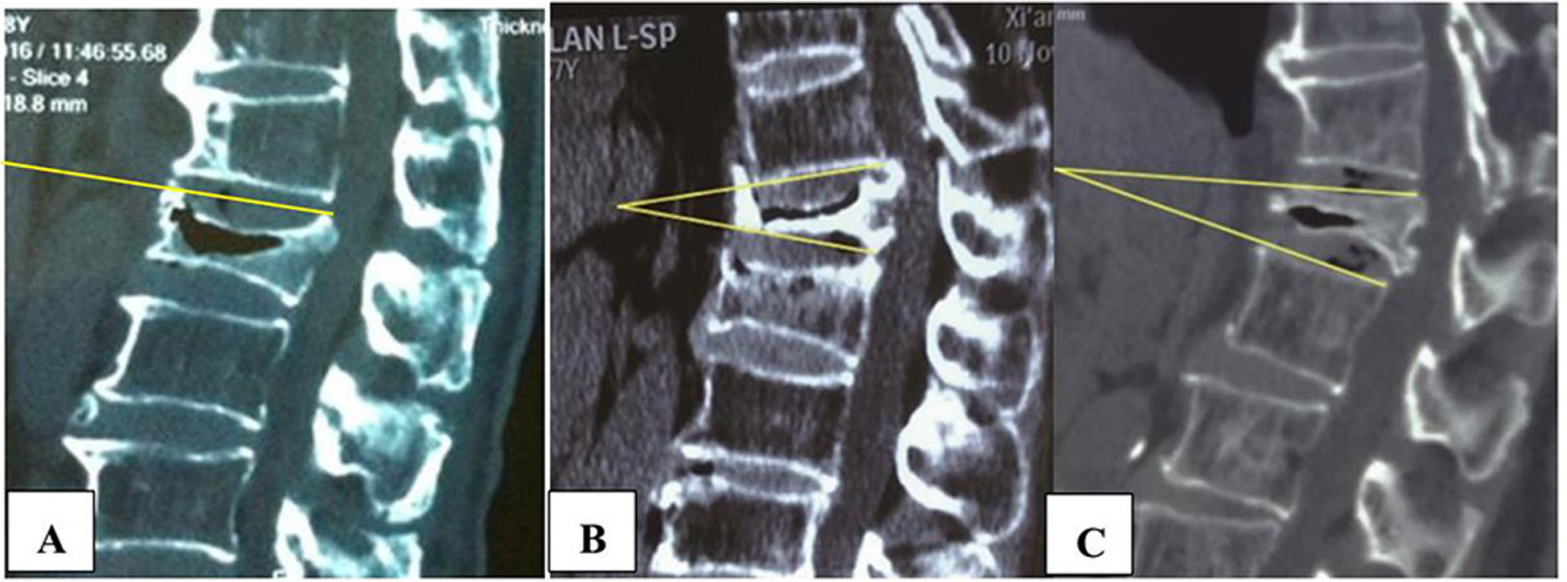
VKA assessment. The angle between the extension lines of upper and lower end plates (**a**). The angle formed by the line connecting the inferior endplates of the adjacent vertebra and the damaged vertebra when the superior endplate of the injured vertebra was collapsed (**b**). The line connecting the superior endplates of the adjacent vertebra and the damaged vertebra when the inferior endplate of the injured vertebra was collapsed (**c**)

Due to vertebral endplate collapse in CSOTF patients, it was difficult to measure the local kyphosis angle accurately. Usually, the injured vertebral kyphosis angle (VKA) refers to the angle between the extension lines of the upper and lower end plates (Fig. 1a). However, it is challenging to measure the VKA in the patients with severe vertebral body wedging (when vertebral collapse exceeds 50%) and hyperplastic osteophyte at the anterior margin of the vertebral body. Thus, we modified the VKA measuring method to facilitate fractured vertebral body stability evaluation (Fig. 1b, c)*.* The difference (D) value between the VKA measured on flexion and extension as visualized with lateral radiography was used to evaluate the stability of the injured vertebra. Considering that some patients aggravate their back pain during activity, the extent of flexion and extension was limited. A supine midsagittal CT scan is a suitable replacement for extension lateral radiography in patients with severe back pain. A VKA *D*-value > 11° was identified as dynamic instability of the injured vertebra. CT and MRI were used to determine spinal stenosis caused by spinal cord bone fragment compression. The degree of local kyphosis was determined using the Cobb kyphosis angle (CKA). The angle formed by the line connecting the superior endplates of the upper adjacent vertebra and the inferior endplates of the lower adjacent vertebra was used to measure the CKA. A CKA > 30° on flexion and extension identified during lateral radiography was determined to be a kyphosis deformity.

### Morphological classification

**Fig. 2:**
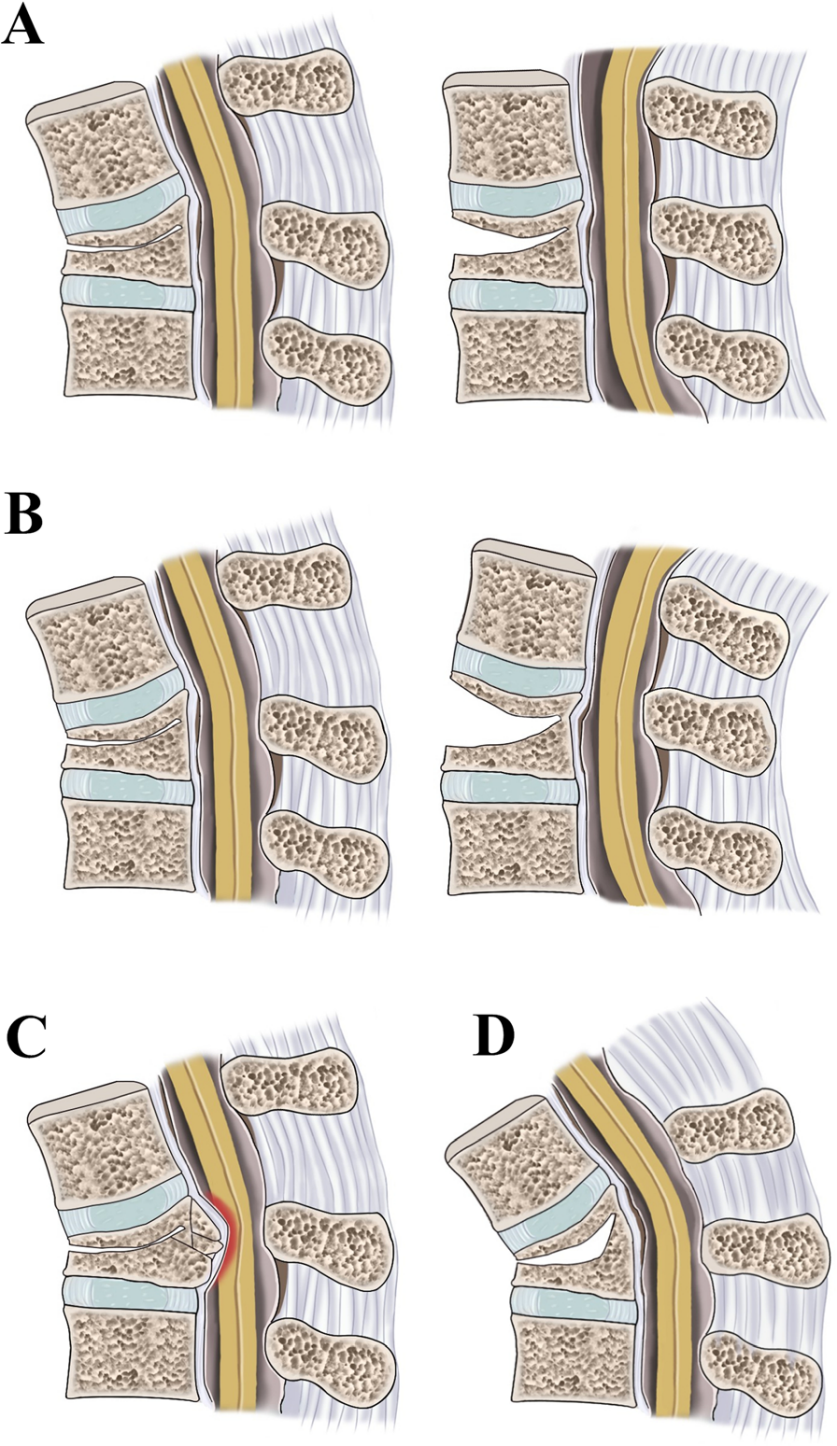
**a** The basic type (type I). **b** The dynamic instability type (type II). **c** The spinal stenosis type (type III). **d** The Kyphosis deformity type (type IV)

After analyzing the imaging data of 368 cases, we revised our new classification system three times before reaching a consensus. *CSOTF* cases were divided into five types based whether the *CSOTF* exhibited dynamic instability, spinal stenosis or kyphosis deformity (Table 1 and Fig. 2). Notably, type I (dynamic stable type) was the main type in our classification.

**Table 1:** A new morphological classification system for chronic symptomatic osteoporotic thoracolumbar fracture

Type	Definition
Type I (dynamic stable type)	It is characterized with the typical radiological changes, such as vertebral vacuum sign, intervertebral cleft, and/or pseudarthrosis. *T*he *D*-value of VKA is less than 11°.
Type II (dynamic unstable type)	The *D*-value of VKA is greater than 11°.
Type III (spinal stenosis type)	MRI reveals backward displacement of the bone fragments that leads to spinal canal stenosis and neurological deficit.
Type IV (kyphotic deformity type)	The CKA is greater than 30° on both flexion and extension lateral radiography.
Type V (mixed type )	Among the 3 aforementioned morphological changes from type II to IV, at least 2 types existed.

### Reliability evaluation

We selected 10 spine surgeons who did not participate in the development of the classification system as evaluators and described the new classification system to them using illustrations in a PowerPoint presentation. All of the evaluators used our system to grade 10 cases of each type of the condition. The unqualified evaluators were re-examined after training to ensure that all evaluators complied with the system. Forty additional cases were graded when three senior experts agreed that all investigators fully understood the system. A second round of grading was performed 1 month later, and the case order was scrambled using a random number generator. All cases included complete imaging data and symptom descriptions.

The reliability of the classification system among different observers and the reproducibility for the same observer on separate occasions were assessed using the Kappa coefficient (*κ*). The coefficients were interpreted using the Landis and Koch grading system [13], which defines the reliability or reproducibility of *κ* values > 0.2 as slight, between 0.2 and 0.4 as fair, between 0.4 and 0.6 as moderate, between 0.6 and 0.8 as substantial, and values > 0.8 as excellent.

### Treatment algorithm

**Fig. 3:**
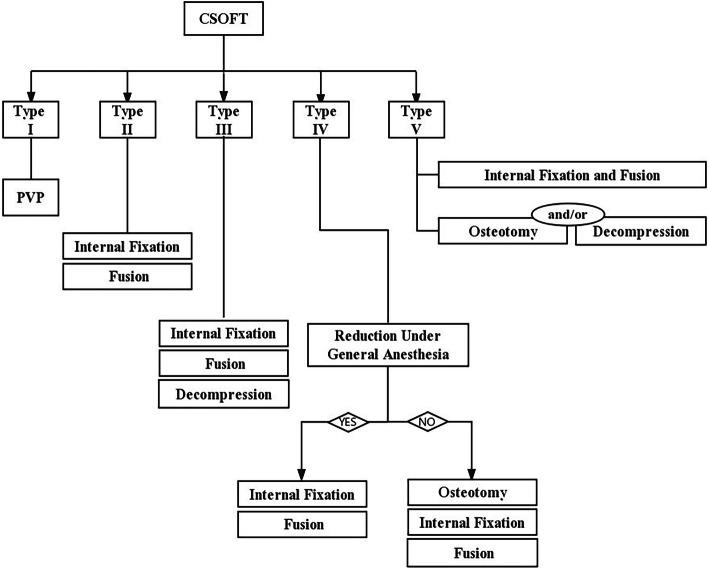
The treatment algorithm for CSOFT

**Fig. 4:**
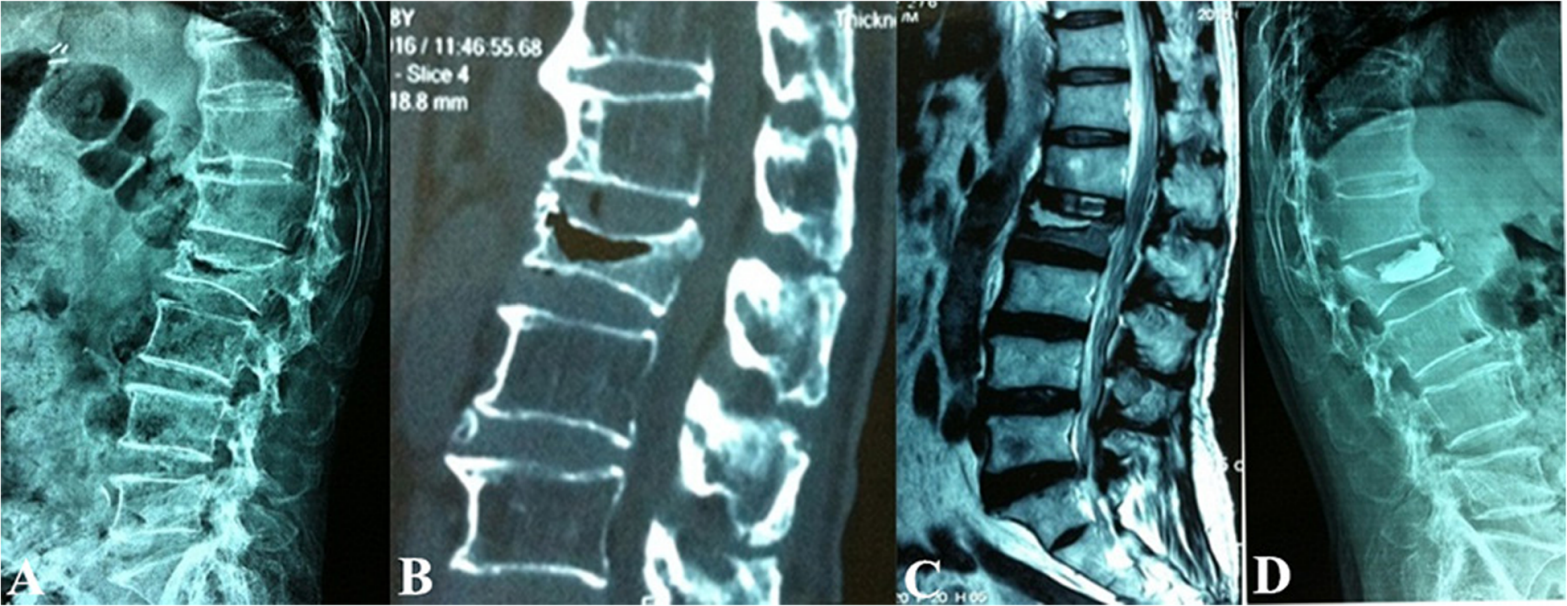
Type I treated with percutaneous vertebroplasty. **a**–**c** The preoperative flexion lateral radiography, supine computed tomography reconstruction view, and MRI, respectively. **d** Postoperative lateral radiography

**Fig. 5:**
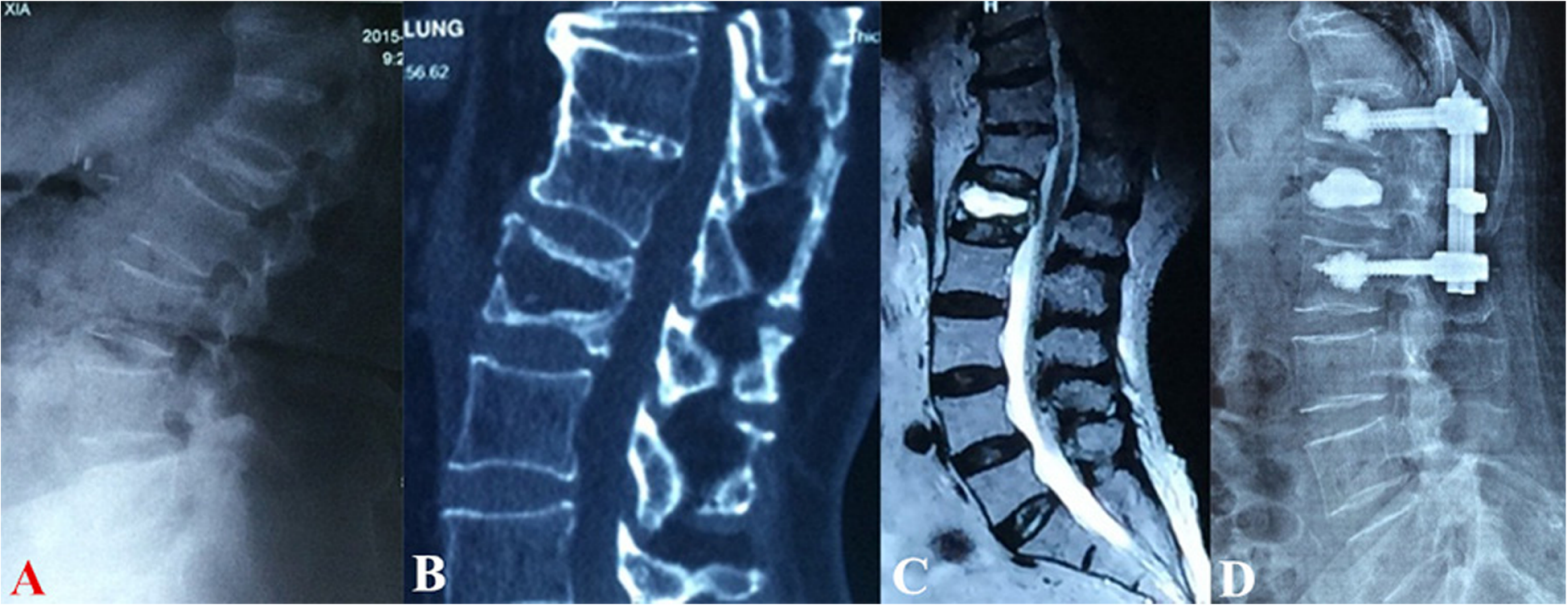
Type II treated using vertebroplasty with short-segment pedicle screw fixation and posterolateral fusion. **a**–**c** The preoperative flexion lateral radiography, supine CT reconstruction view, and magnetic resonance imaging respectively. **d** Postoperative lateral radiography

**Fig. 6:**
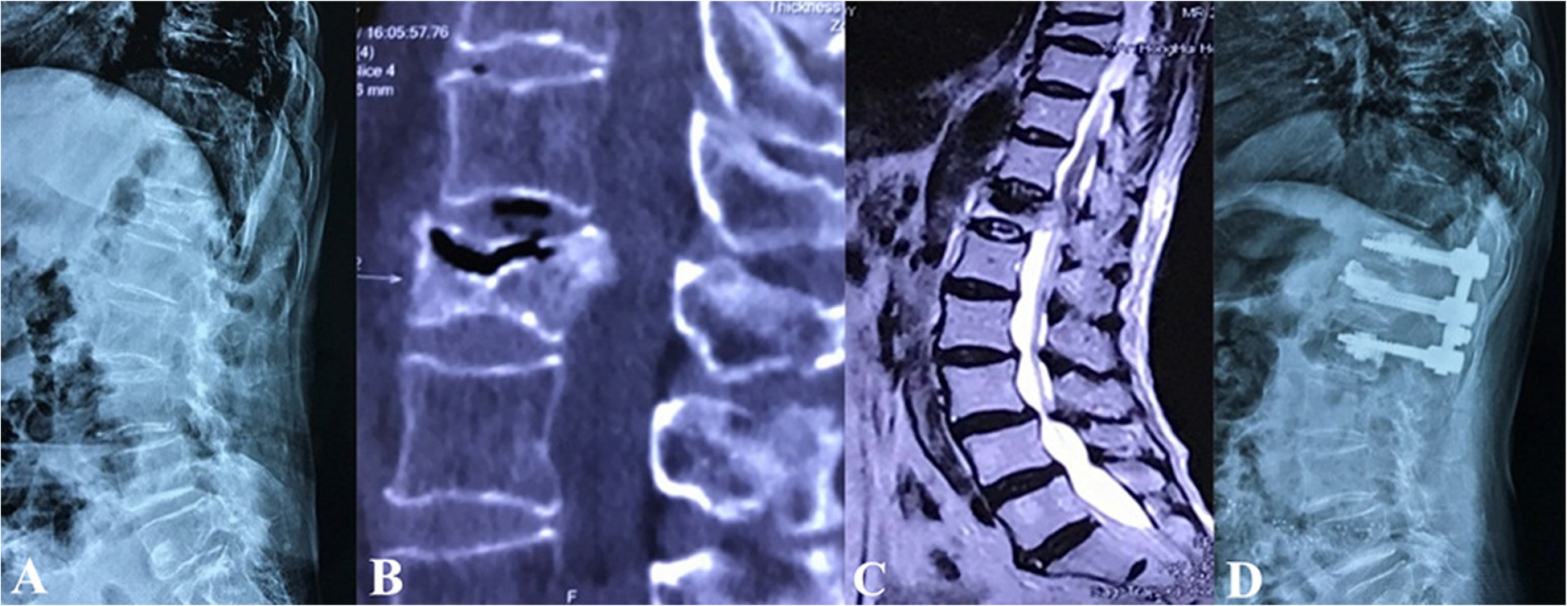
Type III was treated with posterior decompression and bone cement-augmented short-segment pedicle screw fixation and posterolateral fusion. **a**–**c** The preoperative flexion lateral radiography, supine computed tomography reconstruction view, and magnetic resonance imaging, respectively. **d** Postoperative lateral radiography

**Fig. 7:**
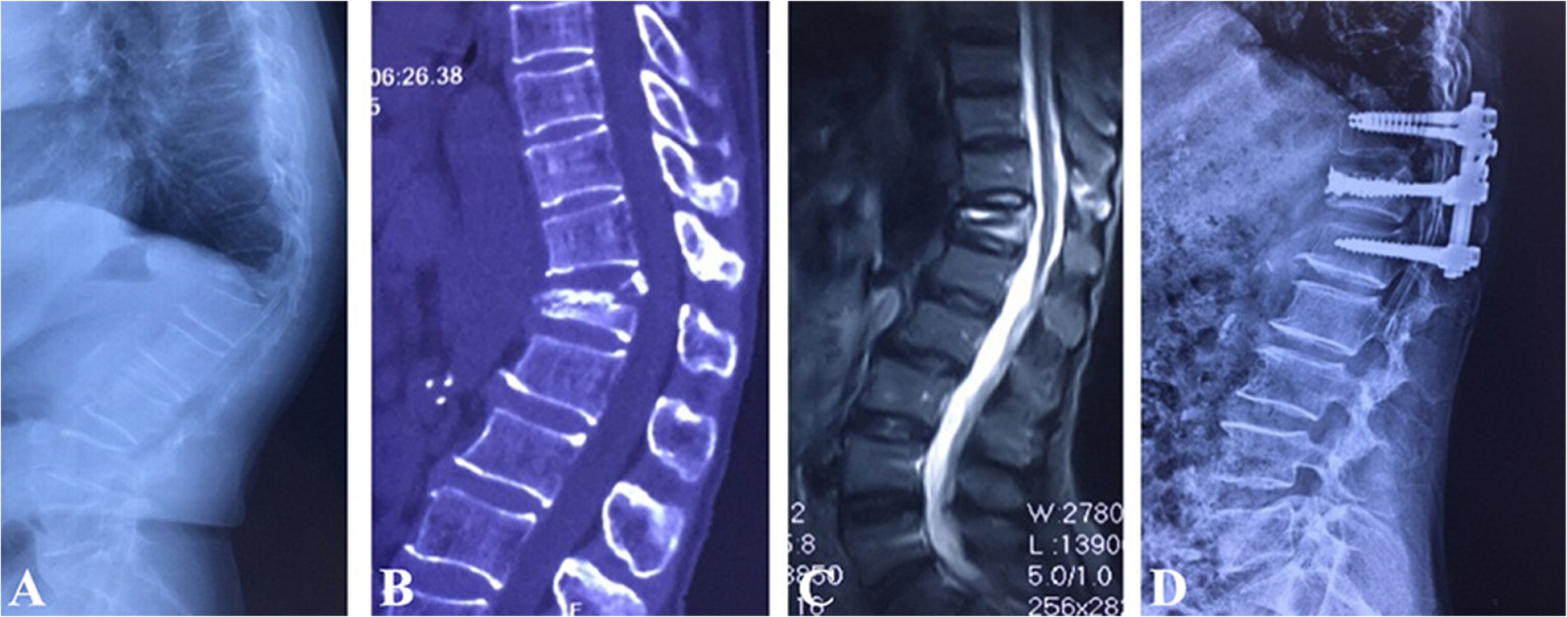
Type IV treated in the over-extending position under general anesthesia combined with short-segment pedicle screw fixation and posterolateral fusion. **a**–**c** The preoperative flexion lateral radiography, supine computed tomography reconstruction view, and magnetic resonance imaging, respectively. **d** Postoperative lateral radiography

**Fig. 8:**
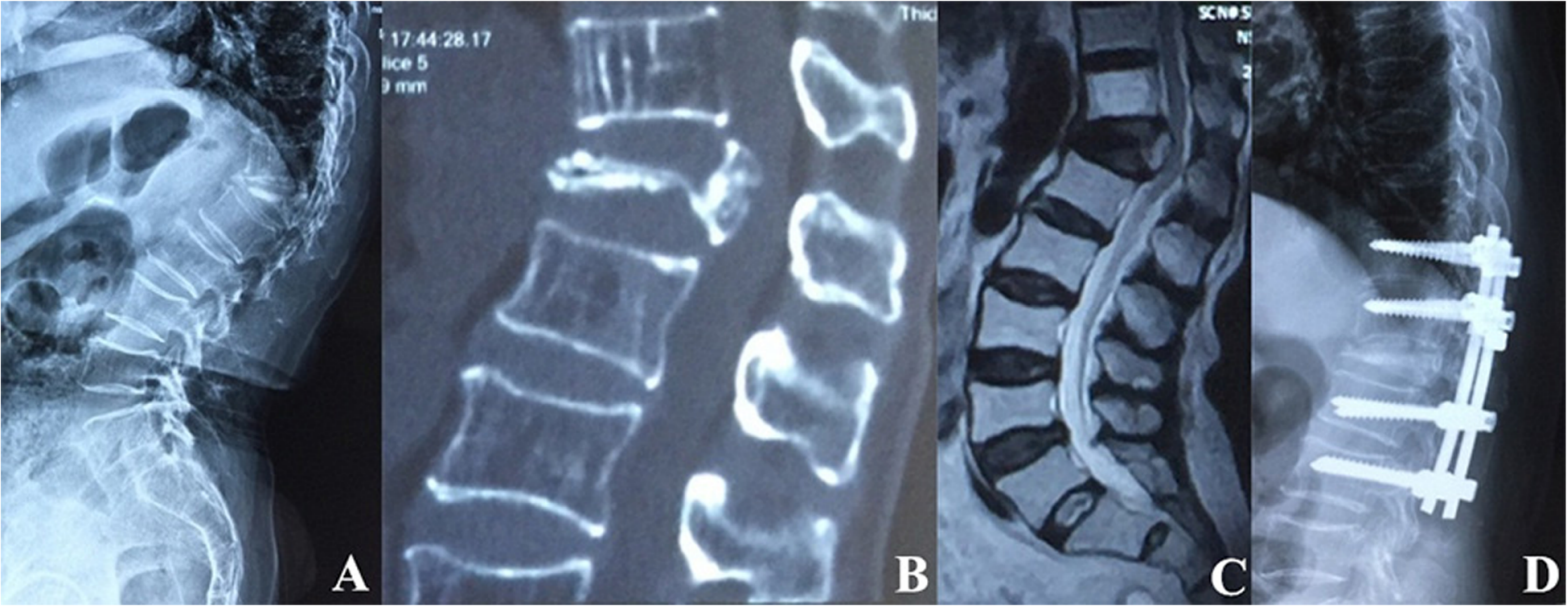
The patient was characterized by “dynamic instability and spinal stenosis and kyphosis deformity”. The surgical approach was posterior decompression and pedicle screw fixation and fusion. **a**–**c** The preoperative flexion lateral radiography, supine computed tomography reconstruction view, and magnetic resonance imaging, respectively. **d** Postoperative lateral radiography

Surgical interventions were indicated for patients with severe pain (VAS score > 4 after oral analgesics) or progressive neurological deficits. Different fracture patterns determine the patient’s symptoms, which subsequently determine the corresponding surgical strategy. The detailed treatment algorithm was summarized in Fig. 3. (1) Type I was the basic type of CSOTF; the injured vertebra was relatively stable, and mild activity of the IVC may be the primary source of back pain. Therefore, we recommended vertebroplasty to relieve pain for this type, which is performed via bone cement injection to eliminate slight motions at the IVC (Fig. 4). (2) Type II typically presents with dynamic instability of the injured vertebra. The apparent activity of pseudarthrosis formed at the fracture region was the cause of back pain. Previous studies have ignored injured vertebrae instability, and patients with this type were treated using vertebroplasty; however, vertebroplasty alone was not suitable for this type. The fibrous tissues and hardened necrotic bone of the inner surface of the IVC obstruct the crosslinking of bone cement with the surrounding cancellous bone, which reduced the bond strength of the cement with the injured vertebra. Therefore, vertebra instability greatly increased the risk of bone cement displacement. Additional posterior instrumentation and posterolateral fusion were considered to be preferable surgeries (Fig. 5). (3) Type III was characterized by spinal stenosis. These patients experienced symptoms of back pain that were usually accompanied by varying degrees of neurological deficits, especially intermittent claudication, which were caused by spinal cord bone fragment compression (the disappearance of the epidural space on MRI and the direct compression of the dural sac by bone fragments). The purpose of surgical treatment was to immediately relieve the compression and stabilize the injured segment. Therefore, decompression, internal fixation, and posterolateral fusion were recommended (Fig. 6). (4) Type IV was characterized by kyphosis deformity, where local kyphosis was > 30°, even in the extended position. Muscle tension of the back triggered by the kyphosis deformity caused persistent back pain, disuse, and muscle atrophy. They may in turn have affected the structure of the posterior tension bands that further aggravate kyphosis. For the patients only with thoracolumbar muscle spasm, muscle relaxation under general anesthesia and nonunion of the vertebral body facilitated the correction of kyphosis with the hyperextension position. Thus, an intraoperative examination should first be performed to verify whether the kyphosis can be corrected in an over-extending position under general anesthesia (Fig. 7). If the CKA is < 30° after hyperextension reduction under general anesthesia, a simple fixation and posterolateral fusion would be adequate. Otherwise, different grades of osteotomy could achieve the correction based on the degree of kyphosis deformity. (5) It is usually the case in type V that one or two pathologies are the main reason for clinical symptoms. Whether decompression or osteotomy should be performed after internal fixation and fusion should be considered primarily in terms of the type of patient that is causing the main symptoms (Fig. 8).

### Clinical outcome evaluation

Clinical outcome evaluation included using the visual analog score (VAS), the Oswestry disability index (ODI), and the American Spinal Injury Association (ASIA) impairment scale. Mean values are presented as the mean ± standard deviation (SD). Student’s *t* test or the analysis of variance (ANOVA) was used to compare differences between measurement data. Rank sum test was used to compare the differences in count data between the two groups. A value of *P* < 0.05 was considered statistical significance. All statistical analyses were performed using GraphPad Prism version 8.0.2 (GraphPad Software Inc., La Jolla, CA, USA).

## Results

### Morphological classification

**Fig. 9:**
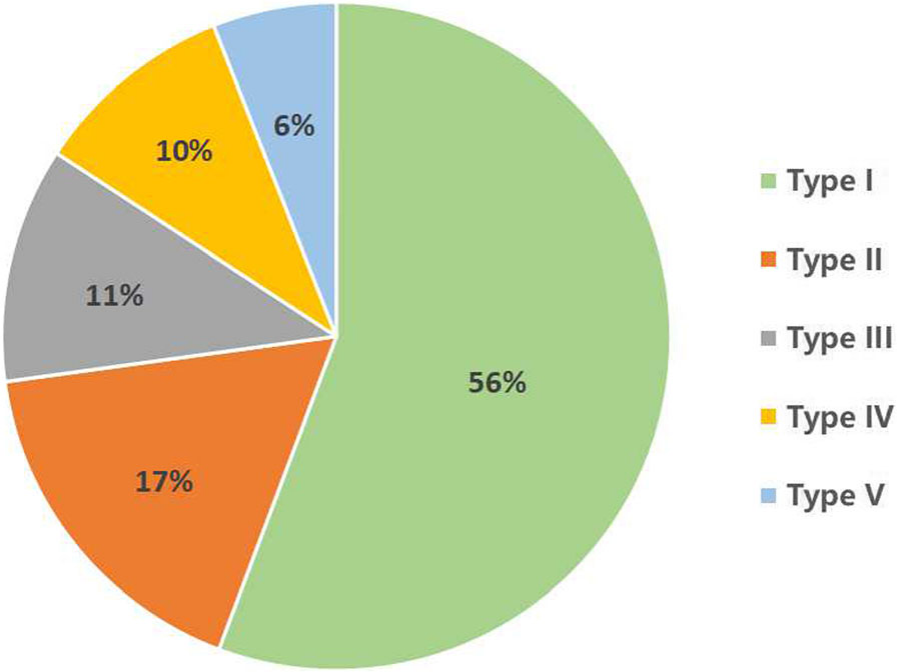
The population distribution for each type

Among the 368 CSOTF patients, the population distribution for each type was presented in Fig. 9. The highest proportion of *CSOTF* was type I (56%), and the lowest proportion of *CSOTF* was type V (6%). The combinations constitute for type V included: type II + type III (*n* = 10, 2.7%), type III + type IV (*n* = 6, 1.6%), type II + type IV (*n* = 4, 1.1%), and type II + type III + type IV (*n* = 2, 0.5%).

### Evaluation of reliability

Ten evaluators performed a total of 800 assessments of 40 cases in two evaluation rounds. The most frequent *CSOTF* type was type III (23.9%), and the least frequently observed type was type V (15.4%) (Table 2).

**Table 2:** Frequency of responses of types

Type	Number	Percent
I	420	52.50
II	140	17.50
III	120	15.00
IV	80	10.00
V	40	5.00
Total	800	100

### Interobserver reliability

The overall interobserver reliability was 0.83, which is considered excellent (Table 3). The interobserver reliabilities for each *CSOTF* type were 0.82 for type I, 0.84 for type II, 0.91 for type III, 0.85 for type IV and 0.71 for type V. These values indicated excellent reliability for types I, II, III, and IV and substantial reliability for type V. The highest agreement was observed for type III (*κ* = 0.91) and the lowest level of agreement was observed for type V (*κ* = 0.71).

**Table 3:** Interobserver reliability

Type	Kappa
I	0.82
II	0.84
III	0.91
IV	0.85
V	0.71
Combined	0.83

### Intraobserver reliability

The average *κ* intraobserver reliability value for all types was 0.85, which is considered excellent reproducibility (Table 4). Six of the 10 evaluators exhibited excellent reproducibility results (*κ* > 0.80) for all types of classification, and none of the evaluators exhibited moderate reproducibility results (*κ* < 0.60).

**Table 4:** Intraobserver reliability

Evaluator	Kappa
1	0.91
2	0.79
3	0.88
4	0.76
5	0.78
6	0.79
7	0.92
8	0.88
9	0.87
10	0.95
Average	0.85

### Outcome evaluation

All patients were followed up for 12–26 months, with an average follow-up of 13.4 ± 1.4 months. The VAS score and ODI of each type were significantly improved at the final follow-up compared with those before surgery (Table 5) (*P* < 0.001, respectively). However, there were no significant differences in the VAS score or ODI between these groups preoperatively and at the final follow-up (*P* > 0.05, respectively). In all patients with neurological impairment, the ASIA grading after surgery was significantly improved compared with that before surgery (Table 6) (*P* < 0.001).

**Table 5:** Comparison of VAS score and ODI of all CSOFT patients between preoperatively and final follow-up

	VAS score					ODI				
	Type I	Type II	Type III	Type IV	Type V	Type I	Type II	Type III	Type IV	Type V
Preoperatively	6.2 ± 1.1	6.6 ± 1.0	6.3 ± 1.2	6.4 ± 1.0	6.7 ± 1.1	63.3 ± 4.6	68.6 ± 3.8	65.1 ± 4.2	66.5 ± 3.9	69.8 ± 3.1
Final follow-up	1.3 ± 0.4	1.4 ± 0.3	1.4 ± 0.2	1.3 ± 0.3	1.5 ± 0.3	31.2 ± 2.2	34.2 ± 2.0	32.5 ± 1.8	31.8 ± 2.2	33.3 ± 1.9

*CSOTF* chronic symptomatic osteoporotic thoracolumbar fracture, *VAS* visual analog score, *ODI* Oswestry disability index

**Table 6:** The ASIA grade of all CSOFT patients at preoperation and final follow-up

		ASIA grade at final follow-up				
Preoperative ASIA grade	*n*	A	B	C	D	E
A	15	\	2	3	7	3
B	22	\	3	7	8	4
C	41	\	\	\	34	7
D	187	\	\	\	9	178
E	103	\	\	\	\	103

*CSOTF* chronic symptomatic osteoporotic thoracolumbar fracture, *ASIA* American Spinal Injury Association

## Discussion

There are several modifications—especially radiological measurement and treatment algorithms—in our classification system. Our results demonstrated that the interobserver reliability of our classification were 0.83, which indicated excellent agreement according to the Landis and Koch grading system. The highest agreement was observed for type III (*κ* = 0.91). One possible reason for this finding is that the morphological characteristics of type III (spinal stenosis) were the most easily identified. The interobserver reliability was lowest for type V, likely because type V is a mixed type *of Kümmell’s disease*. Different evaluators may have identified varying morphological changes in the mixed type, which resulted in inconsistencies among evaluators. However, the *κ* coefficient for type V was 0.71, which indicated excellent agreement. Therefore, this study preliminarily demonstrated that our novel classification system was simple, consistent, and convenient for communication and research.

For the treatment of CSOTF, the following two classifications for Kümmell’s disease are currently recognized by most scholars [9, 14]. Li et al. [9] divided Kümmell’s disease into three stages based on degeneration of adjacent intervertebral discs, the degree of vertebral height loss, and its combination with spinal cord compression; the study by Li et al. described the natural course of Kümmell’s disease. This type of staging of Kümmell’s disease is easy to understand and memorize, but it does not cover all of the fracture patterns found in the condition. Additionally, the Kümmell disease type was classified based on vertebral body height and adjacent intervertebral disc degeneration. However, the correlation between these two factors and the classification and treatment of Kümmell’s disease had not been proved in the literature. The recommended treatment was limited to percutaneous vertebroplasty or open internal fixation, and it was less practical in guiding treatment. In 2013, Patil et al. [14] grouped Kümmell’s disease patients based on the morphological patterns of fracture and proposed surgical options for each group. Kyphosis deformity was first used as a basis for classification, with a deformity > 30° on standing lateral radiography classified as an independent type and treated using pedicle subtraction osteotomy (PSO) and posterior spinal instrumentation. However, in consideration of the possible dynamic instability of the injured vertebral body of some cases, the kyphosis could be reduced or removed with body position changes, and a large invasive treatment, such as PSO, may not be necessary for these patients. Therefore, this classification system incompletely evaluated the kyphosis deformity. Additionally, this classification system did not establish axial instability of type I due to the integrity of the posterior longitudinal ligament complex and the intervertebral joints. This classification system was also based only on 40 patients, which suggests that it was not comprehensive in covering all injury types.

This novel classification system is primarily based on three morphological features of dynamic instability of injured vertebra, spinal stenosis, and kyphosis deformity. Many scholars have described dynamic instability as a unique phenomenon of *Kümmell’s disease* [1, 8, 10, 11], but an accurate evaluation method for dynamic instability was lacking. Dynamic lateral radiography (flexion-extension radiograph) is a classic method for evaluating spinal stability, but it was not feasible *for* some patients with aggravated back pain during activity [15]. A supine midsagittal CT scan could be a replacement for extension lateral radiography in these patients. Considering the vertebral endplate collapse in CSOTF patients, it is not feasible to draw a line on the collapsed end plate for VKA measurement. Therefore, we modified the VKA measuring method. Dissimilar to the VKA applied to evaluate fractured vertebral body stability, CKA reflected the degree of segment kyphosis between the upper and lower adjacent vertebras, which was significant for assessing the necessity of correcting kyphosis. In terms of the age of the patient and osteoporosis, the failure of internal fixation in elderly and osteoporosis patients is quite higher than that in normal adults. Internal fixation should be used with caution in the elderly and in patients with osteoporosis. The VKA *D*-value was measured on flexion and extension as visualized with lateral radiography, which indirectly reflected the stability of intervertebral cleft. At present, the accepted evaluation criterion of lumbar instability is based on the angulation of intervertabral disc in the dynamic sagittal plane. When it is greater than 10°, it is considered lumbar instability [16]. In addition, considering the possible measurement error, we raised the threshold of internal fixation to 11°. The degree of kyphosis in some patients with kyphosis deformity due to the dynamic instability of the injured vertebra may be reduced or disappear in the supine position. Unlike the previous study [14], we defined kyphotic deformity type that the CKA a > 30° on dynamic lateral radiography not only the standing lateral radiography, which was valuable to screen out patients who required corrective operations.

### Treatment algorithm

In this system, type I was the basic type of *CSOTF*, and more than half of the 368 patients exhibited this type (56%). Type II was typical with dynamic instability of the injured vertebra. Tsai et al. [17] reported one case of *Kümmell’s disease* in T12. Preoperative imaging revealed instability of the injured vertebra, and bone cement displacement occurred 1 month after vertebroplasty; this explained why vertebroplasty alone was not sufficient to stabilize the unstable segments and why we recommended additional posterior instrumentation and posterolateral fusion rather than PVP in this type. Type III was characterized by spinal stenosis. Li et al. [9] and Zhang et al. [10] reported posterior decompression, short segmental pedicle screw fixation, and posterolateral fusion combined with vertebroplasty achieved satisfactory results for the treatment of *Kümmell’s disease* with neurological defects, which was consistent with our recommendation. Type IV was characterized by kyphosis deformity. In contrast to previous studies that performed extensive osteotomy in patients with kyphotic deformity [18], we identified patients who required osteotomy through an over-extension reduction test under general anesthesia; this had positive significance for CSOFT’s accurate classification and surgical trauma reduction. Type V was a mixed type that included two or three morphological changes. This type included “dynamic instability and spinal stenosis”, “dynamic instability and kyphosis deformity”, “spinal stenosis and kyphosis deformity”, and “dynamic instability, spinal stenosis and kyphosis deformity”. By identifying patients whose symptoms were primarily caused by spinal stenosis or kyphotic deformity, decompression or osteotomy could be determined after internal fixation and fusion.

### Limitations

Our treatment recommendations were based on the characteristics of each classification as well as our experience, which must be verified in further large-scale prospective clinical trials. It is especially important to balance the benefits and long-term complications when introducing internal implants into osteoporosis patients.

## Conclusion

The new classification system for CSOTF demonstrated excellent reliability in this initial assessment. Treatment algorithm developed based on this classification could greatly improve the clinical efficacy of treatment for CSOFT.

## Data Availability

The datasets generated during the current study are public at the email dingjun.hao@qq.com.

## Abbreviations

OTF: Osteoporotic thoracolumbar fractures
IVC: Intravertebral cleft
CSOTF: Chronic symptomatic osteoporotic thoracolumbar fractures
CT: Computed tomography
MRI: Magnetic resonance imaging
SD: Standard deviations
VKA: Vertebral kyphosis angle
D-value: Difference value
CKA: Cobb kyphosis angle
κ: Kappa coefficient
PSO: Pedicle subtraction osteotomy
